# The Influence of Ply Stacking Sequence on Mechanical Properties of Carbon/Epoxy Composite Laminates

**DOI:** 10.3390/polym14245566

**Published:** 2022-12-19

**Authors:** Raphael Olabanji Ogunleye, Sona Rusnakova, Milan Zaludek, Samuel Emebu

**Affiliations:** 1 Department of Production Engineering, Faculty of Technology, Tomas Bata University in Zlín, Vavrečkova 275, 76001 Zlín, Czech Republic; 2 Department of Automation and Control Engineering, Faculty of Applied Informatics, Tomas Bata University in Zlín, Nad Stráněmi 4511, 76005 Zlín, Czech Republic

**Keywords:** stacking sequence, carbon fibre-reinforced composite, flexural, tensile, impact, composite failure

## Abstract

In this work, the effect of ply stacking sequence of carbon/epoxy laminates subjected to flexural, tensile and impact loading was investigated. Five laminates with different stacking configurations were produced using the hand-laying-up technique. This includes a unidirectional laminate, cross-ply laminates, and quasi-isotropic laminates. Following the autoclave curing process, the responses of the composites to bending, tension and impact force were determined according to ASTM standards, and their corresponding strength, stiffness as well as impact energy were evaluated. Likewise, the flexural failure mode associated with each laminate was characterised using an optical microscope. The unidirectional laminates have higher flexural and tensile strength compared to the cross-ply and quasi-isotropic laminates. Moreover, as a result of material symmetry, the flexural and tensile modulus of symmetric cross-ply laminate improved by 59.5% and 3.97% compared to the unsymmetric counterpart. Furthermore, the quasi-isotropic laminates with absorption energy of 116.2 kJ/m^2^ and 115.12 kJ/m^2^, respectively have higher impact resistance compared to other samples.

## 1. Introduction

In recent years, the use of advanced composite materials in many industries has been growing considerably higher due to their numerous advantages compared to other conventional materials [1,2,3]. Fibre-reinforced polymer composites (FRPs) are considered in an application where a high strength-to-weight ratio is desirable. Moreover, due to their corrosion resistance, relative ease of installation, low density, and high strength, FRPs offer more safety and durability to new and existing engineering structures [4,5]. Thus, industries such as automotive, aerospace, architecture and marine are continuously exploring the potential of this material [6,7]. In civil engineering, fibre-reinforced polymer composites (FRPs) are used in reinforcing bars, sandwich panels and structural retrofitting [8,9]. However, due to the organic nature of the matrix, FRPs are impermeable to vapour, they are flammable and their application on a wet surface at low temperatures is not readily achievable. Additionally, they are susceptible to temperature change; subjection of FRPs to an elevated temperature, as could be witnessed during fire exposure, causes a drastic reduction in their mechanical performance [10]. However, there is growing research interest in overcoming many of these limitations by exploring the usage of inorganic matrices such as fabric-reinforced cementitious matrices [8,11]. Likewise, FRP composites’ brittleness and disastrous failure without sufficient onset warning are generally unacceptable in most engineering applications [12,13,14,15].

Regarding failure, composites are susceptible to damage when they are acted upon by some external force such as tension, compression, and impact load, causing damages that may include delamination, matrix cracking and fibre breakage [16,17,18,19]. In addition, the failure associated with a composite under different loading conditions depends on the type of fibre and resin that constitute the material, layup configuration, thickness and loading speed. Therefore, this failure mode explains the importance of studying composite laminates to achieve a stacking configuration that offers high resistance to deformation and improves tolerance and reliability. 

Lately, the research on the performance of composite structures subjected to different loading conditions has generated much interest. However, only a few studies emphasise the influence of stacking sequence on the behaviour of composite laminates. Caminero, Rodriguez and Munoz [20] studied the effect of stacking sequence on the Charpy and flexural damage of carbon fibre-reinforced polymer composite (CFRP) laminates. It was discovered that the stacking pattern influenced the impact performance and stiffness of the laminate. Mlyniec, Korta, Kudelski and Uhl [21] studied the influence of thickness, stacking sequence and thermal ageing on the behaviour of CFRP laminates. It was revealed that the stacking pattern affects the damping and ageing time of the composites. Grigoriou and Mouritz [22] showed the influence of stacking patterns on the fire resistance of CFRP laminates. Jesthi et al. [23] observed the influence of carbon glass fibre symmetric inter-ply sequence on the mechanical performance of polymer matrix composite. The results showed that the hybrid stacking pattern improved the composite tensile, flexural and impact resistance. Kaboglu et al. [24] studied the impact performances and failure modes of glass fibre-reinforced polymer composite with different curvature and stacking sequences. It was observed that the fibre direction has damping effects on applied force. Singh and Mahesh [25] studied the effect of ply position switching on the impact performance of quasi-isotropic glass fibre-reinforced polymer composite. The results revealed that the position of the lamina influences the impact response. Therefore, the effect of stacking sequence on flexural, tensile and impact damage of composite laminate is yet to be adequately studied.

Thus, in this study, a pre-impregnated carbon/epoxy tape was utilised to produce the laminate composite. Three categories of stacking patterns were considered: unidirectional laminate (${\left[0^{{}^{\circ}}\right]}_{8}$), cross-ply laminates ($\left[0^{{}^{\circ}}/90^{{}^{\circ}}\right]$_4_, $\left[0^{{}^{\circ}}/90^{{}^{\circ}}\right]$_2*s*_), and quasi-isotropic laminates (${\left[0^{{}^{\circ}}/+45^{{}^{\circ}}/90^{{}^{\circ}}/-45^{{}^{\circ}}\right]}_{s},\;{\left[0^{{}^{\circ}}/\pm 45^{{}^{\circ}}/90^{{}^{\circ}}\right]}_{s}$). An autoclave curing process was used to fabricate the laminates. A three-point bending test was conducted to investigate the flexural properties of the laminates. In a similar manner, the tensile properties of the composites were determined on a universal tensile machine, while their impact performances were measured using a pendulum impact tester. The failure modes exhibited by various stacking sequences under flexural loading were analysed by capturing the surface of damaged laminates using an optical micrograph. The practical significance of this study is to provide structural engineers with a better understanding of how the ply stacking pattern of the laminates can be tailored to strengthen structural reinforcement.

## 2. Materials and Methods

### 2.1. Preparation of the Specimen

The carbon/epoxy prepreg tape used in the experiment was sourced from Gurit Composite Company. The prepreg consists of unidirectional (UD), continuous, high-elongation carbon fibres and an epoxy resin system. This material, SE84LV/HEC, with the physicochemical properties listed in Table 1, is commonly used in building yacht hulls [26] and other heavily loaded parts because it possesses high compressive strength. The resin content of the material is 35 ± 3% by weight, while the aerial weight of the fibre is 450 g/m^2^. The width and thickness of the prepreg are 400 mm and 0.440 mm, respectively.

This experiment utilised a 650 mm wide and 900 mm long glass mould to fabricate the composite. The mould was pretreated to overcome cracks and achieve a uniform surface by application of Sealer GP adhesive sourced from Chem-Trend L.P (Howell, MI, USA). This process was conducted by applying a small portion of the adhesive to the surface using a paper towel and then allowing the liquid to react for 15 min before applying the next coating layer. The procedure was repeated three times before the mould was further coated with Chemlease^®^ 2191W releasing agent (also purchased from Chem-Trend L.P, Howell, MI, USA) to ease sample demoulding after curing. Subsequently, UD prepreg tape was cut into sections of approximately 400 mm by 450 mm before laying up each ply with varying stacking patterns into the mould by hand. As shown in Table 2, five laminates were produced, with each sample containing eight layup plies with a total thickness of 3.50 mm. 

The fibre orientation in LM1 is unidirectional, while LM2 and LM3 are cross-ply laminates because the direction of the fibres is at 0^0^ and 90^0^. Similarly, LM4 and LM5 have a quasi-isotropic configuration because the plies are oriented in a manner which may result in behaviour similar to an isotropic material. These stacking patterns help in the evaluation of mechanical properties and modes of failure under different loading conditions. 

**Figure 1:**
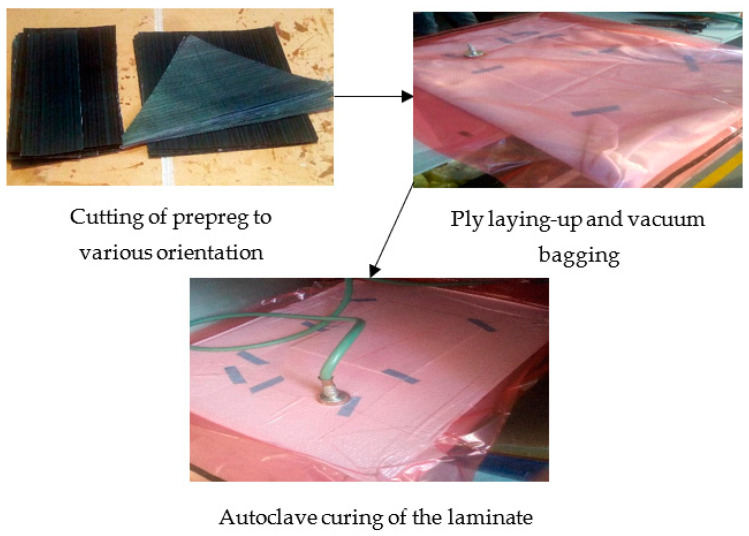
The autoclave manufacturing process of CFRP Laminate.

The laminates were covered with the release film and breather before enclosing them in a vacuum bag, as shown in Figure 1. Afterwards, the setup was heated inside an autoclave from 25 °C to 100 °C at 2 °C/min heating rate and a constant pressure of 6 bar. Then, the process was held under an isothermal condition at this temperature for 180 min before cooling to 25 °C at 3 °C/min. The laminates were evacuated from the autoclave and then demoulded before cutting them into the required dimensions. 

### 2.2. Mechanical Testing

The test specimens were prepared by cutting the laminates along the $0^{{}^{\circ}}$ fibre orientation using a waterjet cutter. Similarly, all the tests were performed at 25 °C and average relative humidity of 30%.

**Figure 2:**
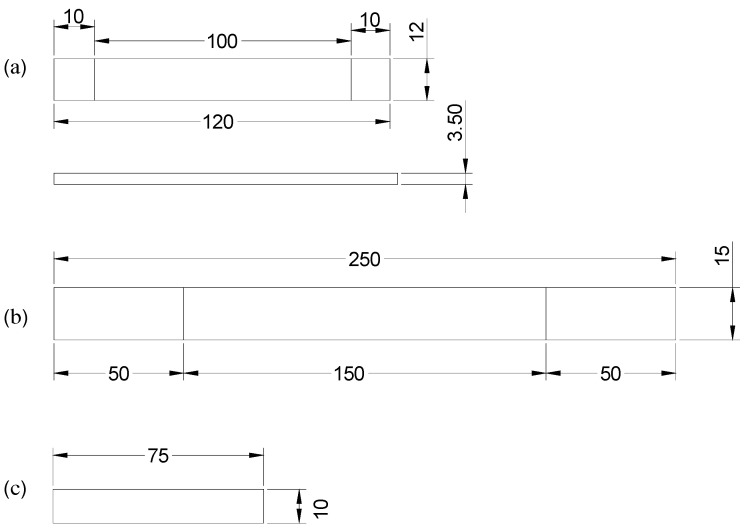
(**a**) Standard three-point flexural specimen as per ASTM D7264. (**b**) Standard tensile specimen following ASTM D3039 recommendation. (**c**) Standard Charpy impact specimen according to ASTM D6110 recommendation.

The flexural test reveals the capability of the composite to resist bending deflection under loading conditions. A three-point bending test was conducted on a Zwick Roell 1456 universal testing machine with a 5 kN load cell at a crosshead speed of 1.0 mm/min according to ASTM D7264 [27]. The specimen with dimensions of 120 mm×15 mm ×3.5 (±0.10) mm and a support span of 100 mm is presented in Figure 2a. Five samples from each laminate were tested to ensure the reliability of the result. The flexural strength and modulus of the materials were determined using Equations (1) and (2).

(1) $$\mathrm{Flexural}\;\mathrm{stress}\;\left(\sigma_{f}\right)=\frac{3\mathrm{FL}}{2\;wt^{2}}$$

(2) $$\mathrm{Flexural}\;\mathrm{modulus}\;\left(E_{f}\right)=\frac{F^{3}m}{4{\;\mathrm{wt}}^{3}}$$

where F is the maximum load (N), L is the support span (mm), w is the width of the specimen (mm), *t* is the thickness (mm), and m is the slope of the tangent to the straight-line portion of the stress-strain plot.

Likewise, the tensile stress ($\sigma_{t}$) and modulus ($E_{t}$) of the composite were measured according to ASTM D3039 [28] on a Shimadzu AGS-XD universal testing machine equipped with a 50 kN load cell, operating at a constant crosshead speed of 2 mm/min. In each scenario, five samples with the dimensions of 250 mm×15 mm ×3.5(±0.1) mm, as shown in Figure 2b, were tested, and the mean values were reported. 

The Charpy impact test was carried out on the samples to measure the amount of energy absorbed under an impact force normal to the laminates. Five specimens per laminate with dimensions of 75 mm ×10 mm×3.5 (±0.1) mm, as shown in Figure 2c, were also tested on a Zwick Roell HIT25P pendulum impact tester with a working capacity of 7.50 J following the ASTM D6110 [29] recommendation.

### 2.3. Optical Microscopy

To analyse the failure mode and crack propagation in the composite with different stacking sequences, the surface layer of the specimen subjected to flexural damage was exposed to an optical microscope using polarised light, and the micrograph images of the samples were captured.

## 3. Results and Discussion

### 3.1. Flexural Properties

**Figure 3:**
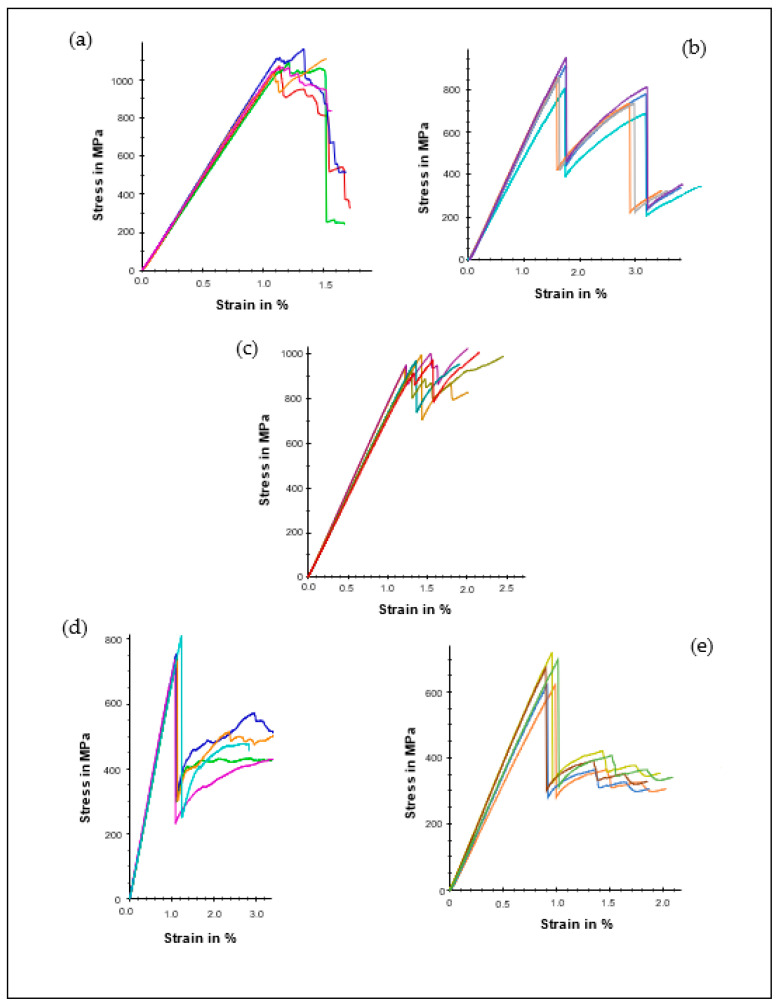
Flexural stress and deformation curves for (**a**) LM1, (**b**) LM2, (**c**) LM3, (**d**) LM4, (**e**) LM5.

The flexural stress-strain relationships are shown in Figure 3. As can be seen, all the samples show a linear relationship up to a particular level of stress. Then, a small nonlinear behaviour close to their ultimate stress was observed. Subsequently, the laminates experience a sudden drop in load due to failure within the internal plies, followed by continuous stress fluctuations at an increasing deformation rate. These results are in agreement with trends reported in previous studies [30,31]. It has been established that material under flexural loading exacts compressive, tensile, and shear stress with associated failure, including delamination, matrix-cracking, fibre breakage, shear splitting, kinking and microbuckling [32,33]. However, influencing material behaviour by manufacturing composite laminates with different stacking patterns may be an essential technique for improving the capability to withstand bending force before failure. 

Among all the configurations studied, LM1 with unidirectional fibre orientation (Table 3) is the most efficient in providing flexural resistance and stiffness, with an average flexural stress $\sigma_{f}$ of 1100 MPa and modulus $E_{f}$ of 98.40 GPa. However, one notable observation in this case (Figure 3a) is that the laminate spontaneous catastrophic failure occurred immediately after the maximum load was attained due to fibre breakage. LM2, a cross-ply laminate, recorded $\sigma_{f}$ of 847 MPa and $E_{f}$ of 46.70 GPa. However, LM2 exhibited higher strain to failure compared to LM1. Moreover, from Figure 3a, it was observed that delamination due to the longitudinal $0^{{}^{\circ}}$ and transverse $90^{{}^{\circ}}$ arrangement of the plies reduces the flexural resistance of LM2 because of the development of transverse matrix cracking, which further increases as the applied load increases. In cross-ply laminate, this phenomenon initiates short delamination, which further coalesces within reinforcing plies, thereby causing instability propagation above and below the midplane of the laminates. Similar behaviour has been reported by Bezazi et al. [34] during flexural testing of a hybrid kevlar/glass cross-ply laminate. The effectiveness of material symmetry can be observed by comparing the flexural properties of LM2 with LM3. 

As shown in Table 3, $\sigma_{f}$ and $E_{f}$ of LM3 increase by 17.9% and 59.5%, respectively. This behaviour could result from a random distribution of carbon fibres across the plies since the lamina geometry above and below the midplane are identical [35,36]. Furthermore, LM4 and LM5, having a balanced and symmetric laminate configuration, presented a flexural behaviour similar to LM3, with 24.5% and 32.5% decreases in $\sigma_{f}$, while the stiffness also decreased by 10.0% and 12.2%, respectively. However, LM4 and LM5 possess high modulus compared to unsymmetrical cross-ply LM2 samples.

Compared to the results obtained by Caminero, Rodriguez and Munoz, the present study obtained an 84.6% increase in flexural modulus for the unidirectional laminate (98.40 GPa). This variation in mechanical properties is due to different processing conditions, physicochemical properties of the materials, manufacturing techniques, the number of ply layups, laminate thickness, and testing conditions. Sudarisman and Davies reported a maximum $E_{f}$ of 61.10 GPa for a unidirectional carbon fibre-reinforced, epoxy laminate fabricated using an autoclave processing technology.

### 3.2. Tensile Properties

**Figure 4:**
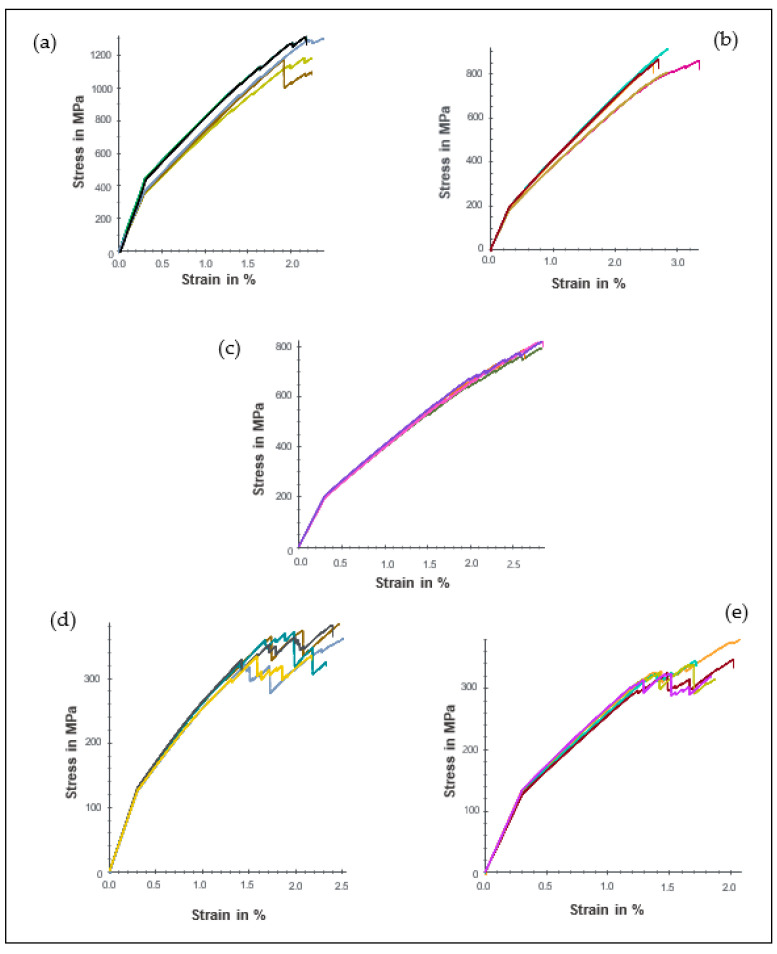
Tensile stress and deformation curves for (**a**) LM1, (**b**) LM2, (**c**) LM3, (**d**) LM4, (**e**) LM5.

The relationship between material stress and strain under tensile loading is presented in Figure 4, while the average tensile stress, $\sigma_{t}$, and modulus, $E_{t}$, are also shown in Table 3. As can be seen, the specimens exhibited a similar trend as in the case of the flexural properties by undergoing a linear relationship in their elastic zone before experiencing a brittle rupture due to the breakage of the fibres. LM1 demonstrated the highest $\sigma_{t}$ of 1220 MPa and $E_{t}$ of 115.0 GPa compared to the other sample. This is due to the longitudinal arrangement of the fibre, which provides high resistance towards tensile loading [31,37,38]. On the other hand, LM2 and LM3 exhibited $\sigma_{t}$ of 859 MPa and 808 MPa, respectively, while their elastic modulus is almost average of LM1. When $0^{{}^{\circ}}$ plies are substituted with $90^{{}^{\circ}}$ plies, as in the case of LM2 and LM3 layup, the number of carbon fibres parallel to the longitudinal loading of the composite decreases, resulting in lower modulus and tensile strength than LM1. Likewise, LM4 and LM5 with multilayer configurations possess lower $\sigma_{t}$ and $E_{t}$ than LM1, LM2 and LM3. Even though there are multiple orientation configurations, one could reasonably deem that transverse and off-axial detachment of the fibre significantly affects the overall resistance of the laminates.

It is also important to note that despite the rearrangement of the stacking pattern of LM4 and LM5, there are no significant differences in their tensile properties since their corresponding $\sigma_{t}$ and $E_{t}$ values, as shown in Table 3, are almost the same.

### 3.3. Charpy Impact Response

**Figure 5:**
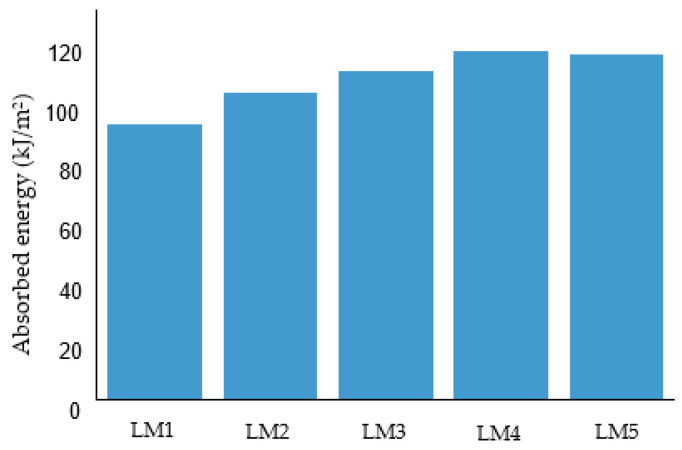
Charpy impact energy of the specimen with different layup sequence.

When an impact force acts upon a composite, energy is released, with part of it utilised in elastic deformation, while excess energy is dissipated through various mechanism that leads to the failure of the material [39,40,41,42]. Therefore, the degree of damage due to an impact force depends on the amount of energy absorbed by the laminate. Figure 5 shows the result of the Charpy impact test of the specimens in terms of the energy absorbed. As can be seen, the unidirectional laminate, LM1, absorbed lower energy (92.10 kJ/m^2^) compared to the multidirectional, LM2 (102.21 kJ/m^2^), LM3 (109.40 kJ/m^2^), LM4 (116.2 kJ/m^2^) and LM5 (115.12 kJ/m^2^) laminates.

This characteristic exhibited by LM1 could be due to brittle fibre breakage and matrix cracking that are less obvious in other laminates (LM2, LM3, LM4 and LM5) with a multidirectional layup sequence. The $90^{{}^{\circ}}$ layers in the cross-ply structure prevented the matrix crack from spreading across the layer thickness since the mechanism of loading during impact is an out-of-plane transverse loading. This allowed LM2 to absorb more energy during impact than LM1. This behaviour has been reported in previous works on Charpy impact loading of composite [43,44,45,46,47]. Moreover, it is observed that LM4 and LM5, quasi-isotropic laminates, exhibited average absorption energy that is higher than cross-ply laminates (LM2, LM3) due to the inheritance of impact performance of both angle ply and unidirectional laminates, thus improving their impact performance.

### 3.4. Failure Analysis

**Figure 6:**
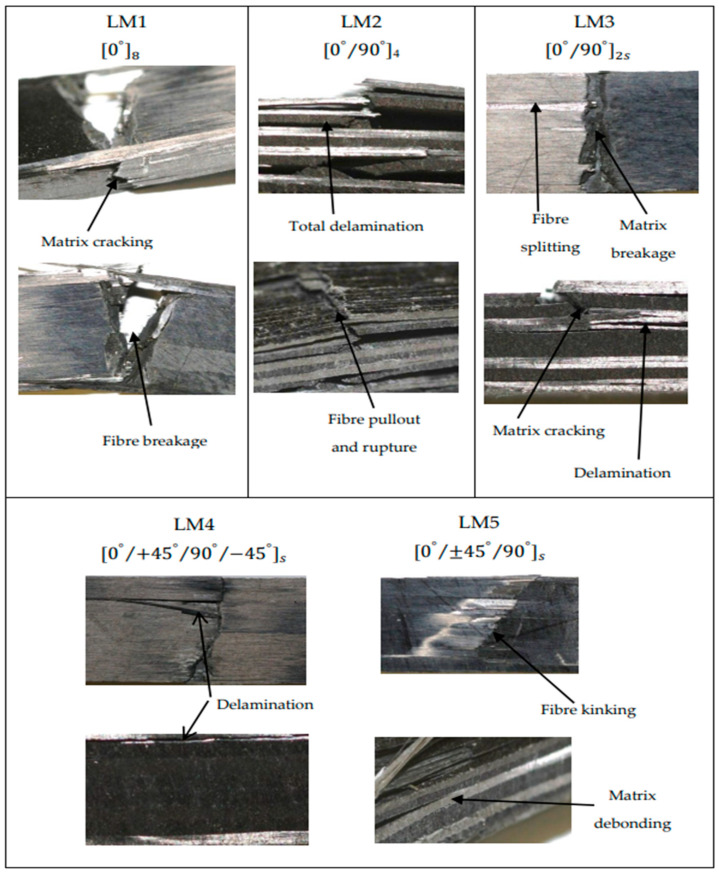
Micrograph image of the fractured specimen under flexural loading condition.

An essential factor determining the type of failure exhibited by a composite laminate is the ply stacking sequence that also determines the orientation of the fibre [20,48,49]. Other common influences include the nature of material composition and the type of load applied. Figure 6 illustrates the optical micrograph of the specimen under flexural loading. As can be seen for the unidirectional laminate (LM1) case, the sample failed by transverse cracking of the matrix and fibre breakage, resulting in a drastic fall of the flexural stress, as shown in Figure 2a. On the other hand, LM2 exhibited high interlayer delamination between $0^{{}^{\circ}}/90^{{}^{\circ}}$ stacking configuration, as revealed in Figure 6. This confirmed the unbalanced stress distribution in Figure 2b. Moreover, fibre rupture along the $0^{{}^{\circ}}$ longitudinal direction was noticed. Likewise, LM3 displayed a similar mode of failure as LM2 but minimal interlayer delamination due to the symmetry and balanced nature of the cross-ply laminate. Additionally, LM4 and LM5 exhibited lower interlayer delamination than other specimens, while fibre kinking and matrix debonding were also observed.

## 4. Conclusions

This study examined the effect of stacking configuration of CFRP laminates subjected to flexural, tensile and impact loading. An optical micrograph of the damaged laminate surface was used to explain the failure mode of the composite. The following conclusions can be drawn based on the results obtained:

1. In flexural mode, unidirectional laminate demonstrated the highest flexural strength and modulus. However, it undergoes premature catastrophic failure.
2. The unidirectional laminate (LM1) recorded the maximum tensile strength and modulus. This results from the alignment of the laminate ply orientation with the principal loading direction, which gives rise to the orthogonal behaviour of the composite. The carbon fibre also impacted the overall tensile properties due to the strong adhesive force between the carbon fibre and epoxy matrix, which provide high tensile properties to the laminate.
3. The multidirectional quasi-isotropic laminates (LM4 and LM5) recorded the highest impact resistance due to high-impact energy.
4. Microscopic analysis indicated that the laminates failed under flexural loading in typical matrix cracking, fibre pull-out, matrix debonding and delamination modes.

Further research is required to study the compression and shear properties, which can play a vital role in further analysing the internal and interface failure of the laminates. 

## Figures and Tables

**Table 1:** Physicochemical properties of SE84LV/HEC carbon fibre/epoxy system [26].

Properties	SE84LV/HEC
Fibre density (g/cm^3^)	1.8
Resin content (%)	35 ± 3
Fibre volume fraction (%)	55
Fibre aerial weight (g/m^2^)	400
Prepreg thickness (mm)	0.440

**Table 2:** Schematic representation of the laminate ply stacking sequences.

Laminate Samples				
LM1	LM2	LM3	LM4	LM5
${\left[0^{{}^{\circ}}\right]}_{8}$	${\left[0^{{}^{\circ}}/90^{{}^{\circ}}\right]}_{4}$	${\left[0^{{}^{\circ}}/90^{{}^{\circ}}\right]}_{2s}$	${\left[0^{{}^{\circ}}/+45^{{}^{\circ}}/90^{{}^{\circ}}/-45^{{}^{\circ}}\right]}_{s}$	${\left[0^{{}^{\circ}}/\pm 45^{{}^{\circ}}/90^{{}^{\circ}}\right]}_{s}$
$0^{{}^{\circ}}$	$0^{{}^{\circ}}$	$0^{{}^{\circ}}$	$0^{{}^{\circ}}$	$0^{{}^{\circ}}$
$0^{{}^{\circ}}$	$90^{{}^{\circ}}$	$90^{{}^{\circ}}$	$+45^{{}^{\circ}}$	$+45^{{}^{\circ}}$
$0^{{}^{\circ}}$	$0^{{}^{\circ}}$	$0^{{}^{\circ}}$	$90^{{}^{\circ}}$	$90^{{}^{\circ}}$
$0^{{}^{\circ}}$	$90^{{}^{\circ}}$	$90^{{}^{\circ}}$	$-45^{{}^{\circ}}$	$-45^{{}^{\circ}}$
$0^{{}^{\circ}}$	$0^{{}^{\circ}}$	$90^{{}^{\circ}}$	$-45^{{}^{\circ}}$	$-45^{{}^{\circ}}$
$0^{{}^{\circ}}$	$90^{{}^{\circ}}$	$0^{{}^{\circ}}$	$90^{{}^{\circ}}$	$90^{{}^{\circ}}$
$0^{{}^{\circ}}$	$0^{{}^{\circ}}$	$90^{{}^{\circ}}$	$+45^{{}^{\circ}}$	$+45^{{}^{\circ}}$
$0^{{}^{\circ}}$	$90^{{}^{\circ}}$	$0^{{}^{\circ}}$	$0^{{}^{\circ}}$	$0^{{}^{\circ}}$

s; symmetry.

**Table 3:** Average flexural and tensile properties of the CFRP with different stacking sequences.

Sample	Flexural		Tensile	
	$\sigma_{f}\left(\mathrm{MPa}\right)$	$E_{f}\left(\mathrm{GPa}\right)$	$\sigma_{t}\left(\mathrm{MPa}\right)$	$E_{t}\left(\mathrm{GPa}\right)$
LM1	1100 (37.6)	98.40 (3.07)	1220 (54.40)	115.00 (3.32)
LM2	847 (131)	46.70 (0.93)	859 (37.70)	62.90 (0.86)
LM3	999 (22.3)	74.50 (3.20)	808 (43.10)	65.40 (1.00)
LM4	754 (32.7)	67.00 (1.98)	374 (15.00)	42.70 (1.22)
LM5	674 (43.80)	65.40 (3.05)	362 (24.50)	44.10 (1.05)

Standard deviation is shown in the bracket.

## Data Availability

The data presented in this study are available on request from the corresponding author.
